# Prevalence of SARS-CoV-2-specific antibodies in a sample of the Lithuanian population-based study in Spring 2023

**DOI:** 10.1016/j.heliyon.2024.e29343

**Published:** 2024-04-12

**Authors:** Martynas Simanavičius, Indrė Kučinskaitė-Kodzė, Snieguolė Kaselienė, Skirmantė Sauliūnė, Dainius Gudas, Ligita Jančorienė, Rūta Jasinskienė, Astra Vitkauskienė, Rasa Žūtautienė, Aurelija Žvirblienė, Mindaugas Stankūnas

**Affiliations:** a Institute of Biotechnology, Life Sciences Center, Vilnius University, Saulėtekio al. 7, LT-10257, Vilnius, Lithuania; b Department of Health Management, Lithuanian University of Health Sciences, Tilžės g. 18, LT-47181, Kaunas, Lithuania; c Clinic of Infectious Diseases and Dermatovenerology, Institute of Clinical Medicine, Faculty of Medicine, Vilnius University, M. K. Čiurlionio g. 21, LT-03101, Vilnius, Lithuania; d Faculty of Public Health, Lithuanian University of Health Sciences, Tilžės g. 18, LT-47181, Kaunas, Lithuania; e Department of Laboratory Medicine, Lithuanian University of Health Sciences, Eivenių g. 2, LT-50161, Kaunas, Lithuania; f Department of Environmental and Occupational Medicine, Lithuanian University of Health Sciences, Tilžės g. 18, LT-47181, Kaunas, Lithuania

**Keywords:** COVID-19, SARS-CoV-2, Lithuania, Anti-N IgG, Seroprevalence

## Abstract

**Objectives:**

Despite positive trends in SARS-CoV-2 epidemiology, seroprevalence surveys remain an important tool for estimating the magnitude of the COVID-19 pandemic. This study aimed to investigate the prevalence of IgG antibodies against SARS-CoV-2 nucleocapsid (N) and spike (S) proteins in a sample of the Lithuanian population (N = 517) and evaluate how the pattern of seropositivity correlates with the levels of SARS-CoV-2 infection and vaccination.

**Methods:**

Study participants (aged 18–88 years) filled in the questionnaire self-reporting their demographic-social variables, health status, and SARS-CoV-2-related status. The anti-S and anti-N IgG levels were estimated using a microarray ELISA test.

**Results:**

After several pandemic waves and vaccination campaign, the seroprevalence of SARS-CoV-2-specific IgG in the analyzed sample was 97.87 % by March–May 2023. We determined the 96.91 % prevalence of anti-S and 58.03 % prevalence of anti-N IgG. The majority of study participants (71.18 %) had hybrid immunity induced by vaccination and SARS-CoV-2 infection. 20.3 % of study participants were anti-N IgG positive without reporting any previous symptoms or a positive SARS-CoV-2 test. A decline of anti-N IgG positivity within 9 months after infection was observed.

**Conclusions:**

This study demonstrates high total seroprevalence in March–May 2023 in all age groups indicating a widely established humoral immunity against SARS-CoV-2 in Lithuania.

## 1 Introduction

The COVID-19 pandemic has been a devastating public health challenge in many countries. There have been 1321328 confirmed cases of COVID-19 with 9691 deaths in Lithuania [1]. The critical point in the management of the COVID-19 pandemic has been the availability of COVID-19 vaccines. A massive population vaccination began on June 30, 2021, reaching a 67 % vaccination rate by June 2023 [1]. Despite the present positive trends in Lithuanian and global COVID-19 epidemiology [2], sero-surveillance studies remain an important tool for estimating the magnitude of the COVID-19 pandemic and monitoring its dynamics. Data from such surveys can contribute to a better understanding of the extent of SARS-CoV-2 infection and further planning of infection control and prevention policies. Lithuania's first national SARS-CoV-2 seroepidemiologic survey was conducted in August–September 2020 [3]. It was found that the seroprevalence was 1.4 %, indicating higher real prevalence of the virus across the population than officially estimated.

It is well known that previous SARS-CoV-2 infection results in the development of both anti-nucleocapsid (anti-N) and anti-spike (anti-S) antibodies, while COVID-19 vaccines induce only anti-S antibodies [4]. Therefore, periodic serologic studies can show antibody responses to SARS-CoV-2 infection and vaccination and indicate potential groups at risk of infection in the general population by incorporating extensive questionnaire data on the presence of other diseases, lifestyle, experienced symptoms, and different socio-demographic factors. Unfortunately, such nationwide studies are scarce [[5], [6], [7], [8], [9]].

This study aimed to investigate the prevalence of anti-N and anti-S IgG in blood serum specimens in a sample (N = 517) of the Lithuanian population-based study and compare the pattern of seropositivity with the status of self-reported SARS-CoV-2 infection and vaccination.

## 2 Materials and methods

### 2.1 Study design and procedures

This is a cross-sectional seroprevalence study on the presence of anti-N and anti-S immunoglobulins G (IgG) in a sample of the Lithuanian population. The study was carried out in March–May 2023. Inclusion criteria for individual participants were: more than 18 years old, living in Vilnius and Kaunas cities or districts, and having no mental disabilities. The required sample size was calculated with an expected prevalence of 50 %, margin error of 5 %, confidence level of 95 %, and total population of municipalities (N = 1050173) [10]. The minimum recommended sample size for the survey was 385. Participants were enrolled in two ways: randomly selected and using convenient, non-random sampling (Fig. S1). A random selection of individuals was made by the Lithuanian State Enterprise Centre of Registers. Study participants were selected to represent the population in terms of age and gender. They received the invitations by regular mail. In total, out of 6143 invited individuals, 210 agreed to participate. Convenient non-random sampling was performed by inviting all interested individuals to participate in the study. Inclusion criteria were applied the same as for the random sample. Invitations were published in different mass-media channels. The number of recruited participants was 307. Thus, the total sample size was 517.

Participants were asked to visit the designated health care unit, where they provided a written consent, filled in the questionnaire and had their capillary blood drawn. They reported gender, date of birth, place of residence, education, occupation, height, weight, health status, and COVID-19-related experience.

### 2.2 Serological testing

Capillary blood specimens were collected using the Microvette 200 Z-Gel (Sarstedt, Germany) system for capillary blood specimen collection to separate blood serum by centrifugation for 5 min at 10,000×*g* (Fig. S1). Serum specimens were tested using the “Coronavirus IgG MaE” test (UAB Imunodiagnostika, Lithuania). The test is based on the use of recombinant SARS-CoV-2 S glycoprotein ectodomain expressed as secreted trimeric protein in mammalian (CHO) cells (UAB Baltymas, Lithuania) and recombinant SARS-CoV-2 N protein expressed in *Saccharomyces cerevisiae* (UAB Baltymas). The test's microarray images were acquired and analyzed using a sciREADER CL2 reader and its software (SCIENION GmbH, Germany). The anti-S IgG results were expressed as binding antibody units per ml (BAU/ml). The anti-S IgG results are standardized according to the First WHO International Standard for anti-SARS-CoV-2 immunoglobulin (human) (NIBSC code: 20/136). The anti-N IgG results were semi-quantitative and expressed in relative units (RU). The anti-S IgG cut-off value is 13 BAU/ml while the anti-N IgG cut-off value is 1 RU, and the sample is considered positive if the result is greater or equal to this value. According to the manufacturer, the test performance characteristics for the detection of anti-S IgG are 100 % (95 % confidence interval (CI): 91.2–100 %) sensitivity and 98.9 % (95 % CI: 95.7–99.9 %) specificity, and for detection of anti-N IgG are 92.7 % (95 % CI: 80.1–98.5 %) sensitivity and 97.6 % (95 % CI: 94.0–98.5 %) specificity. The specimen is considered seropositive if either anti-S or anti-N IgG or both are positive by the test.

### 2.3 Definitions for groups of infection-induced and vaccination-induced immunity

Study participants were divided into non-overlapping groups of induced immunity: only infection, only vaccination, hybrid immunity, and no immunity (Table 1). These categories were defined by the self-reported SARS-CoV-2 infection and vaccination status and the results of serological testing performed within this study.

**Table 1:** Definitions of participant groups based on self-reported characteristics and seropositivity.

Category	Self-reported vaccination	Self-reported infection	Anti-N IgG positive	Anti-S IgG positive	Total (%/n) (N = 517)
Infection-induced immunity	No	Yes and/or anti-N IgG	Yes or no and/or anti-S IgG	Yes and/or anti-N IgG	10.64/55
Vaccination-induced immunity	Yes	No	No	Yes	17.21/89
Hybrid immunity	Yes	Yes	Yes or no	Yes or no	71.18/368
No immunity	No	No	No	No	0.97/5

### 2.4 Statistical analysis

Data were analyzed using the Statistical Package for Social Sciences (IBM SPSS Statistics) version 29.0 and GraphPad Prism 9.5.1. Qualitative variables were reported as absolute and relative frequencies (%). CI was calculated by the modified Wald method [11]. The normality of quantitative variables was tested by the Kolmogorov-Smirnov test (all variables were not normally distributed). Quantitative variables were expressed by the mean and standard deviation (mean ± SD). Differences between groups were tested using a chi-squared test (three or more groups), Fisher exact test, or Mann–Whitney test (two groups). Significance was defined by a P value of 0.05.

## 3 Results

### 3.1 COVID-19 pandemic in Lithuania and study group description

**Fig. 1:**
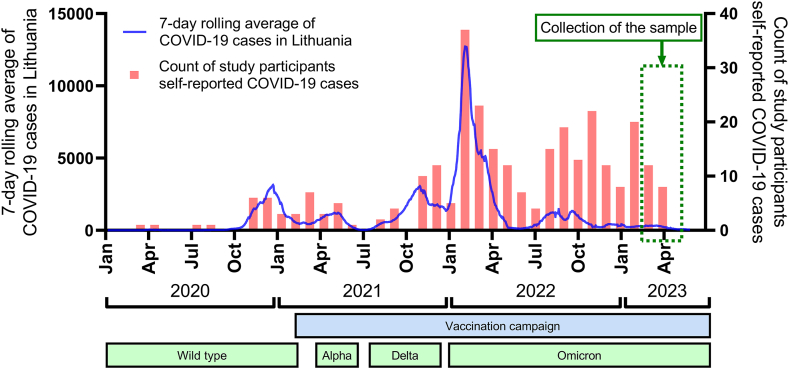
Comparison of the dynamics of the COVID-19 pandemic in Lithuania and the cases of self-reported SARS-CoV-2 infection of study participants. Infection cases are represented as a 7-day rolling average of SARS-CoV-2 infections (based on the WHO Coronavirus (COVID-19) dashboard [12], left Y axis) represented as a blue line. The number of study participants who self-reported COVID-19 cases (right Y axis) in the timescale is represented as red columns. A period of collection of the sample and serum specimens analyzed in this study is marked with a green dashed line. The period of the vaccination campaign and predominant SARS-CoV-2 variants are shown as light blue and light green text boxes, respectively.

The development of the COVID-19 pandemic in Lithuania is shown in Fig. 1. Serum specimens analyzed in this study were collected after the Omicron subvariants infection waves. At the time of serum specimen collection, 42.3 % of the Lithuanian population had been confirmed to be infected with SARS-CoV-2 one or more times, 69.7 % had been vaccinated with at least one dose, and 67 % had been fully vaccinated [1]. The majority of self-reported infection cases among study participants occurred during the dominance of the SARS-CoV-2 Omicron subvariants.

A description of the study sample (N = 517) and study sample groups by the vaccination from SARS-CoV-2 infection status is provided in Table S1. The study sample consisted of 67.5 % of females. More than half of the study participants were of middle age (40–64 years). A total of 457 (88.4 %) individuals were vaccinated. Comparative analysis of the different sample groups by vaccination status showed statistically significant associations between education, employment levels, chronic respiratory disease, and vaccination status. A significantly higher proportion of the vaccinated study participants had a university education and were employed compared to those not vaccinated. Also, a significantly higher proportion of nonvaccinated study participants self-reported chronic respiratory system disease than vaccinated ones.

Self-reported SARS-CoV-2 infection characteristics by the vaccination status are provided in Table S2. Comparison of self-reported SARS-CoV-2 infection characteristics with vaccination status showed that a statistically significantly higher proportion of vaccinated individuals either did not report previous SARS-CoV-2 infection confirmed by PCR test or did not report previous SARS-CoV-2 infection not confirmed by any test, compared to not vaccinated. Also, a statistically significantly smaller proportion of vaccinated study participants reported symptoms during the last SARS-CoV-2 infection, such as frequent breathing, shortness of breath, and diarrhea, compared to not vaccinated ones.

### 3.2 Prevalence of antibodies specific to SARS-CoV-2 S and N proteins

All study participants (N = 517) were tested for the presence of antibodies specific to SARS-CoV-2 S and N proteins. Anti-S IgG was detected in 501 (96.91 %), while anti-N IgG was detected in 300 (58.03 %) participants (Table 2). The total observed seroprevalence was 97.87 % (95 % CI: 96.18–98.86 %). The results of the anti-N and anti-S IgG positivity are similar when stratified by gender or age. Anti-N IgG was determined in 54.17 % of men, and 59.89 % of women, while anti-S IgG was determined in 95.24 % of men and 97.71 % of women. The total seroprevalence in men and women was 97.02 % (95 % CI: 93.03–98.91 %) and 98.28 % (95 % CI: 96.21–99.30 %), respectively. In age groups of 18–39, 40–64, and more than 65 years old, the prevalence of anti-N IgG was 52.71 %, 60.29 %, and 58.62 %, respectively. In these age groups, the percentages of anti-S IgG positivity were 96.12 %, 97.43 %, and 96.55 %, respectively, and the total seroprevalence was 97.67 % (95 % CI: 93.08–99.51 %), 98.53 % (95 % CI: 96.14–99.56 %), and 96.55 % (95 % CI: 91.18–98.94 %), respectively.

**Table 2:** Demographic characteristics of participants and the seroprevalence of anti-S and anti-N IgG in the study group. Numbers are n (%) unless noted differently. The seroprevalence is estimated based on any anti-SARS-CoV-2 IgG positivity.

Categories		Total	Gender		Age groups, y		
			Men	Women	18–39	40–64	65+
**All participants**	Number of participants	517	168 (32.50)	349 (67.50)	129 (24.95)	272 (52.61)	116 (22.44)
	Anti-S positive	501 (96.91)	160 (95.24)	341 (97.71)	124 (96.12)	265 (97.43)	112 (96.55)
	Anti-N positive	300 (58.03)	91 (54.17)	209 (59.89)	68 (52.71)	164 (60.29)	68 (58.62)
	Seroprevalence, % (95 % CI)	97.87 (96.18–98.86)	97.02 (93.03–98.91)	98.28 (96.21–99.30)	97.67 (93.08–99.51)	98.53 (96.14–99.56)	96.55 (91.18–98.94)
**Hybrid immunity**	Number of participants	368 (71.18)	118 (32.07)	250 (67.93)	100 (27.17)	193 (52.45)	75 (20.38)
	Anti-S positive	367 (99.73)	118 (100.0)	249 (99.6)	100 (100.0)	192 (99.48)	75 (100.0)
	Anti-N positive	259 (70.38)	79 (66.95)	180 (72.00)	58 (58.00)	143 (74.09)	58 (77.33)
	Seroprevalence, % (95 % CI)	100.0 (98.75–100.0)	100.0 (96.21–100.0)	100.0 (98.18–100.0)	100.0 (95.56–100.0)	100.0 (97.65–100.0)	100.0 (94.16–100.0)
**Only infection**	Number of participants	55 (10.64)	17 (30.91)	38 (69.09)	13 (23.64)	30 (54.55)	12 (21.82)
	Anti-S positive	45 (81.82)	11 (64.71)	34 (89.47)	9 (69.23)	25 (83.33)	11 (91.67)
	Anti-N positive	41 (74.55)	12 (70.59)	29 (76.32)	10 (76.92)	21 (70.00)	10 (83.33)
	Seroprevalence, % (95 % CI)	89.09 (77.82–95.26)	82.35 (58.16–94.62)	92.11 (78.48–98.00)	84.62 (56.54–96.90)	90.00 (73.58–97.34)	91.67 (62.47–99.99)
**Only vaccination**	Number of participants	89 (17.21)	31 (34.83)	58 (65.17)	15 (16.85)	48 (53.93)	26 (29.21)
	Anti-S positive	89 (100.0)	31 (100.0)	58 (100.0)	15 (100.0)	48 (100.0)	26 (100.0)
	Anti-N positive	0 (0.00)	0 (0.00)	0 (0.00)	0 (0.00)	0 (0.00)	0 (0.00)
	Seroprevalence, % (95 % CI)	100.0 (95.04–100.0)	100.0 (86.91–100.0)	100.0 (92.57–100.0)	100.0 (76.14–100.0)	100.0 (91.15–100.0)	100.0 (84.76–100.0)
**No immunity**	Number of participants	5 (0.97)	2 (40.00)	3 (60.00)	1 (20.00)	1 (20.00)	3 (60.00)
	Anti-S positive	0 (0.00)	0 (0.00)	0 (0.00)	0 (0.00)	0 (0.00)	0 (0.00)
	Anti-N positive	0 (0.00)	0 (0.00)	0 (0.00)	0 (0.00)	0 (0.00)	0 (0.00)
	Seroprevalence, % (95 % CI)	0.00 (0.00–48.91)	0.00 (0.00–70.98)	0.00 (0.00–61.75)	0.00 (0.00–83.25)	0.00 (0.00–83.25)	0.00 (0.00–61.75)

By comparing the self-reported characteristics of SARS-CoV-2 vaccination and the seropositivity, it was found that the more times the participants were vaccinated, the lower anti-N IgG seropositivity was observed. The percentage of anti-N IgG-positive individuals after 1, 2, 3, and 4 vaccinations was 83.3 %, 61.9 %, 55.1 %, and 41.7 %, respectively (p = 0.036, Table S3). In a group of participants who self-reported to have had a previous SARS-CoV-2 infection, the proportion of anti-N IgG-positive individuals was significantly higher than in a group of participants who did not self-report previous infection (61.3 % vs. 51.2 %, p = 0.030, Table S3). Anti-N IgG seropositivity in groups of participants that self-reported SARS-CoV-2 infection after vaccination (n = 200), before vaccination (n = 53), or without vaccination (n = 40) were 63.5 %, 56.6 %, and 67.5 %, respectively (p > 0.05, data not shown). The more times a previous SARS-CoV-2 infection was confirmed by rapid antigen test, the higher percentage of anti-N IgG-positive individuals was determined (p = 0.033, Table S3). When comparing the groups with or without self-reported household exposure to SARS-CoV-2, higher anti-N IgG positivity was observed in the exposed individuals (62.6 % vs. 53.2 %, p = 0.031, Table S3).

### 3.3 Comparison of antibody to SARS-CoV-2 prevalence by induced immunity type

Anti-SARS-CoV-2 IgG positivity was investigated in groups of individuals by infection or/and vaccination-induced immunity (Table 1). The majority of study participants (71.18 %) belong to the hybrid immunity group, while 10.64 % and 17.21 % of participants had only infection-induced and only vaccination-induced antibodies, respectively. Only 0.97 % of individuals were seronegative. The highest seroprevalence (100 %) was observed in vaccination-induced and hybrid immunity groups, while in the infection-induced immunity group, the seroprevalence was 89.09 % (p < 0.0001, Table 2). The highest anti-S IgG positivity was in vaccination-induced (100 %) and hybrid (99.73 %) immunity groups, while in the infection-induced immunity group, it was 81.82 % (p < 0.0001). By analyzing anti-N IgG positivity in participants with hybrid immunity stratified by age, significant differences of 58 %, 74.09 %, and 77.33 % positivity in 18–39, 40–64, and 65+ years of age groups, respectively, were observed (p = 0.0056). Proportions of anti-N IgG positive individuals with hybrid immunity, who did not self-report SARS-CoV-2 infection, are 15 %, 26.43 %, and 34.7 % of participants in 18–39, 40–64, and 65+ years old groups, respectively (p = 0.0097).

### 3.4 The dynamics of anti-SARS-CoV-2 IgG positivity

**Fig. 2:**
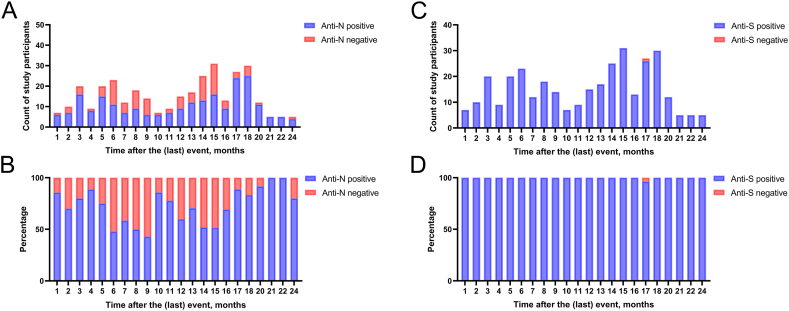
Anti-SARS-CoV-2 antibody persistence in participants with hybrid antibody-mediated immunity. In panel (A) count of anti-N IgG positive and negative individuals, (B) proportion percentage of anti-N IgG positive and negative individuals, (C) count of anti-S IgG positive and negative individuals, and (D) proportion percentage of anti-S IgG positive and negative individuals is shown. Timepoints with less than 5 individuals are excluded; all data is visualized in Fig.S4.

In further analysis, anti-N and anti-S IgG positivity in differently induced immunity groups by the time after the last notified antibody-inducing event was compared. Trends of long-term persistence of anti-N or anti-S IgG in the infection-induced immunity and vaccination-induced immunity groups were undeterminable (Figs. S2 and S3, respectively). In the hybrid immunity group, there were several time points with more than 5 individuals (Fig. S4). It was found that anti-N IgG positivity declines up to 9 months after the last event (Fig. 2). One month after infection, anti-N IgG positivity is 85.71 %. It declines to 42.86 % at month 9 (Fig. 2). Anti-N IgG positivity at months 10–38 shows no clear tendency. Anti-S IgG positivity remains high during 24 months since the last notified event in the hybrid immunity group (Fig. 2). The same pattern applies to the vaccination-induced immunity group (Fig. S3), whereas in the infection-induced immunity group, there are time points with lower anti-S IgG positivity (Fig. S2).

## 4 Discussion

Serologic tests evaluating the SARS-CoV-2 seroprevalence provide a detailed understanding of the extent of SARS-CoV-2 infection in the population. The information from serosurveys helps to better understand virus spread and its impact on different populations. In the current study, we investigated the anti-SARS-CoV-2 seropositivity in a sample of population-based study by measuring anti-N and anti-S IgG, thus showing the developed antibody-mediated immunity after vaccination, infection, or both. Anti-N IgG is generated after infection and acts as a valuable marker for identifying individuals with a mild or asymptomatic infection, especially in cases when routine COVID-19 testing was not applied [13,14]. Anti-S IgG generation is an indication of either vaccination, infection, or both [15]. Combinations of anti-N IgG and anti-S IgG seropositivity may help to confirm information gathered by questionnaires, identify asymptomatic infections, and estimate the actual situation of antibody-mediated immunity to SARS-CoV-2. Thus, measurement of at least these immune markers in serum samples allows to perform an informative serosurvey. The other abundantly expressed virus structural protein, membrane (M) protein also elicits a strong humoral and cellular immune response in COVID-19 patients [16]. It has been reported that some individuals (0 %, 4.2 %, or 5.29 % of tested samples) have had cross-reactive antibodies to SARS-CoV-2 in blood specimens collected in the pre-pandemic period [[17], [18], [19]]. These antibodies have been likely generated against some common epitopes located in regions of N, S, and non-structural ORF1-encoded proteins with high sequence homology among human coronaviruses [20]. It is known that circulating SARS-CoV-2-specific CD8^+^ and CD4^+^ T cells in blood samples of COVID-19 patients exhibit immunodominant patterns for M, S, and N proteins. The reactivity of CD8^+^ T cells with the M protein in blood samples of COVID-19 patients was identified to be as strong as the reactivity with the S protein [21]. However, antibodies against the M antigen are not widely measured in serologic diagnostics. Much less is known about the humoral response to other SARS-CoV-2 proteins, therefore, they are not exploited as markers in SARS-CoV-2 serology.

During the COVID-19 pandemic in Lithuania, the highest SARS-CoV-2 infection incidence rate was observed during the Omicron predominance period [1]. In our study sample, the majority of participants self-reported the previous infection during this period (the year 2022). Massive PCR testing ended in Lithuania in May 2022. Therefore, many individuals may have had an unconfirmed SARS-CoV-2 infection. In our study, blood specimens were collected in March–May 2023, which is a year after the end of massive PCR testing. This explains the high number of self-reported infection cases in our study sample and suggests that the prevalence of SARS-CoV-2 infection might be underestimated. Therefore, the determination of anti-N IgG is an alternative way to estimate the prevalence of SARS-CoV-2 infection, especially in Autumn 2022 and Winter 2023, as anti-N IgG persists within 6 months [6].

The study design to invite participants was highly challenging. A mixed model for inviting participants was applied. The response rate of randomly selected individuals was only 3.4 %, whereas in 2020 it was 14.1 % [3]. Therefore, a non-random sampling of interested individuals was applied. This indicates the challenging nature of collecting an appropriate random sample by invitation at the end of the pandemic. It was estimated that non-random sampling is associated with a higher participation rate compared to random sampling [22]. However, we have acknowledged the higher risk of response bias in applying the non-random sampling method, as agreement to participate in a study could be influenced by several factors that in turn are related to the likelihood of previous exposure: socioeconomic status, trust in government or the medical profession, and many others [23]. Nevertheless, results from other studies suggest, that under the most penalizing scenarios of selection of participants, the population prevalence estimate changes minimally compared to non-random sampling [24]. There are SARS-CoV-2 seroprevalence studies, which apply both types of sampling – random and non-random. For instance, such a design was applied in Iceland and a small variation in the prevalence estimates between open invitation and random selection recruitments was observed [25]. Moreover, representativeness is not considered an essential factor for seroprevalence surveys in establishing the validity of the association between serological status and the incidence of infection [9]. As the main aim of our survey was to evaluate the pattern of seropositivity at the end of the COVID-19 pandemic and compare it with the status of self-reported SARS-CoV-2 infection and vaccination, we believe that the chosen sampling method is relevant and that it is highly unlikely that the results would change in any meaningful way given the sample size or sampling procedure used. While studies based on a random sampling remain epidemiologically the most valuable, considering their limitations, especially low response rates, it would be worthwhile to pursue alternative strategies in continuing seroepidemiological research in the future. For instance, conducting serological investigations on blood plasma or serum samples collected in biobanks or healthcare institutions could be considered. Many recent SARS-CoV-2 seroepidemiological surveys are using blood donor samples [6,26,27]. On the other hand, random sampling by invitation was successful in some populations [8,9]. Sampling by invitation allows the collection of additional information through questionnaires, which is not available when testing residual specimens from blood donors. Alternatively, serological studies could be performed within a targeted sample, such as the communities of healthcare workers, scientists, or patients. In the presence of a high prevalence of illness, this approach would reliably reflect trends in the broader population.

There are a few studies investigating the seroprevalence after the waves of Omicron subvariants in Europe. Our results showed that the seroprevalence is 97.87 % by March–May 2023. This observed seroprevalence has increased dramatically compared to the previous result (1.9 %) [3]. Here, we determined the 96.91 % and 58.03 % prevalence of anti-S and anti-N IgG, respectively. There were no comparable differences in data stratified by age or gender, or by comparing participants with and without comorbidities as well as stratifying data by age (data not shown). The lower prevalence of anti-N IgG (27.3–80.2 %) compared to widespread anti-S IgG (84.1–99 %) induced by vaccination and infection was also observed in several seroprevalence studies performed in other countries [5,6,8,9,[26], [27], [28], [29]]. The 84.1 % prevalence of anti-S IgG and 48.6 % of anti-N IgG were detected in Slovenia during March–June of 2022 [6] The 93.8 % prevalence of anti-S IgG and 72.4 % of infection-induced anti-N IgG were detected in a population-based serosurvey in April–June 2022 in Switzerland [8]. Another study in Switzerland reported a 98.8 % prevalence of anti-S IgG and 36.7 % of anti-N IgG in June 2022 [29]. A global meta-analysis study estimated a total seroprevalence of 95.9 % and a prevalence of anti-N antibodies of 47.9 % in Europe in March 2022 [28]. Another study conducted in April–June 2022 in Portugal reported a 95 % prevalence of anti-S IgG and 27.3 % of anti-N [27]. During the same period in Spain, a 92.7 % prevalence of anti-S IgG and a 58.9 % prevalence of anti-N IgG were found [5]. These studies were performed at different times during the COVID-19 pandemic and show a gradual increase of the seropositivity. However, it is difficult to compare these results with our data as our study was performed at the time the COVID-19 pandemic wanes. In Finland, a 99 % prevalence of anti-S IgG and 54 % of anti-N IgG were observed during October–December 2022 [9]. In Wales, above 99 % prevalence of anti-S IgG and 80.2 % of anti-N IgG was detected in blood donors in November 2022 [26]. The prevalence of anti-SARS-CoV-2 IgG in our study sample is similar to a recent study in Finland, which observed a 99 % prevalence of anti-S IgG and 54 % of anti-N IgG in October–December 2022 [9]. It is not surprising as there is only 3–5 months difference in sampling time. A higher anti-S IgG-positivity is explained by the generation of antibodies against S protein after both vaccination and infection. After SARS-CoV-2 infection, the estimated time to 50 % seronegativity of anti-S IgG is more than 2 years, while that of anti-N IgG is less than 1 year [30]. In addition, after natural infection, the persistence of anti-S IgG for 13 months was shown [31], while anti-N IgG levels decrease after 3 months up to 6 months [32]. Even though this anti-N IgG positivity decline is described by others [6], the mechanisms behind this are not fully explained.

Seroprevalence data should be interpreted keeping in mind several factors including environmental factors, exclusion of children from the study sample, and various comorbidities influencing antibody formation. In the Northern Hemisphere compared to Southern Hemisphere SARS-CoV-2 spreads faster as the lower the temperature, the more infection cases are detected [33]. An increase of infection cases was reported in the temperature range of 0–17 °C [34]. These temperatures are common for the long period lasting through Autumn, Winter, and Spring seasons in the Baltic region where this study was performed. There are limited data on the seroprevalence rates in children. In our study, children were not included in the sample. Seroprevalence rates in children during Omicron predominance were investigated in a few previous studies. A study in Germany investigated children aged 2–17 years and showed an 85.5 % prevalence of anti-S IgG and a 67.2 % prevalence of anti-N IgG in July–October 2022 [35]. In England, a 97.2 % prevalence of anti-S IgG and 86.7 % of anti-N IgG was detected in September 2022 in children aged 1–17 years [36]. Varying seroprevalence patterns in adults and children and between countries could be explained by different vaccination and infection rates in children. Individuals with comorbidities comprise another specific group to evaluate anti-SARS-CoV-2 seroprevalence. We did not find any significant differences in seropositivity in the comorbidities groups. Similar observations were reported from Brazil in October 2020–February 2021 [37]. On the contrary, in unvaccinated infected children, a slight decrease in the antibody response was observed in those with comorbidities in the study performed in Argentina in March 2020–July 2021 [38]. Anti-SARS-CoV-2 seroprevalence should be investigated further to evaluate the impact of comorbidities on antibody-mediated immunity.

In our analysis of seroprevalence stratified by self-reported status of SARS-CoV-2 vaccination and infection, anti-N IgG prevalence decreases with every subsequent vaccination dose. This was observed before [5,6]. It might be explained by the precautious attitude of vaccinated individuals to avoid SARS-CoV-2 infection. In our study, no differences in anti-N IgG seropositivity were observed in groups of participants who self-reported infection either before vaccination, after vaccination, or without vaccination. In another study, it was shown that anti-N IgG levels were lower in individuals who got vaccinated and then infected compared to those who were infected and then got vaccinated [39]. In contrast, the same study demonstrated that anti-S IgG levels are higher in those vaccinated and then infected compared to those infected and then vaccinated [39]. In our study, anti-S IgG seropositivity remains high in all groups regardless of the sequence of antibody-inducing events (data not shown). Higher anti-N IgG positivity in individuals who self-reported previous SARS-CoV-2 infection confirmed by rapid antigen test was determined. Participants who felt symptoms might have performed the SARS-CoV-2 rapid antigen test at home as self-testing was very popular throughout the Omicron waves, and symptomatic infection induced longer-lasting anti-N response [32]. Part of the study participants (20.3 %) were anti-N IgG positive without reporting any previous symptoms or a positive SARS-CoV-2 test, which might be explained by asymptomatic infection cases. This suggests that the serologic anti-N IgG test has the potential to reveal the real SARS-CoV-2 incidence rate [9,28]. A rapid increase of anti-N seroprevalence in the general population in Finland was observed during the beginning of the Omicron wave [40]. Our study revealed higher anti-N seropositivity in individuals with self-reported household exposure as compared to unexposed. Close contact with infected individuals is a way SARS-CoV-2 spreads [41]. Thereby, anti-N IgG testing may be a valuable tool in the estimation of SARS-CoV-2 incidence rate in exposed yet asymptomatic individuals.

We grouped the study participants by infection, vaccination-induced, or hybrid immunity types. In this study, 71.18 % of individuals had hybrid immunity, which is higher compared to 51 % in Finland [9]. Age-stratified proportions of participants with hybrid immunity also showed a tendency to decline by age [9]. The high percentage of participants with hybrid immunity could be fated by breakthrough infections of the Omicron variant [42], which caused the formation of hybrid immunity in vaccinated individuals. The highest total seroprevalence was found in vaccination-induced and hybrid immunity groups compared to lower seroprevalence in the infection-induced immunity group. The analysis of anti-S IgG showed that all participants with vaccination-induced and hybrid immunity were seropositive, while 81.82 % of participants with infection-induced immunity were seropositive. Vaccination induces high anti-S IgG levels that decrease within 4–6 months and remain well above the cut-off for a long period [43]. Hybrid immunity is formed by both vaccination and infection. This explains anti-S IgG positivity observed in vaccination-induced and hybrid immunity groups, while post-infection anti-S IgG levels declined in time. A similar tendency was observed previously as 95.7 % and 99.1 % of participants with only vaccination-induced immunity and hybrid immunity were anti-S IgG positive, respectively, while 77.2 % of individuals in the infection-induced immunity group were seropositive [6].

The analysis of anti-N IgG prevalence revealed 74.55 % and 70.38 % seropositivity in the infection-induced and hybrid immunity groups, respectively. A 71.6–72.4 % anti-N IgG positivity was observed in the infection-induced immunity group [6,8], while a 57.5 % prevalence of anti-N IgG was detected in the hybrid immunity group [6]. In our study, the anti-N IgG prevalence in the infection-induced immunity group follows other studies, while anti-N IgG positivity in the hybrid immunity group is higher than reported by others. The sampling period of this study is later, which may influence higher anti-N IgG positivity in the hybrid immunity group as there was more time for individuals to get infected.

Age-stratified anti-SARS-CoV-2 IgG positivity rates vary in infection-induced, vaccination-induced, and hybrid immunity groups. In the hybrid immunity group, an age-related increase in anti-N IgG positivity was found. This could be explained by observed 15 %, 26.43 %, and 34.7 % proportions of participants with hybrid immunity, who were anti-N IgG positive, but did not self-report SARS-CoV-2 infection, in age groups of 18–39, 40–64, and 65+ years, respectively. These unconfirmed by PCR or rapid antigen tests, allegedly asymptomatic infections were probably more recent, reflecting the late phase of the Omicron wave. Anti-N IgG positivity may indicate the overlooked infections in older individuals with hybrid immunity that were more prevalent in our study sample. Furthermore, this result demonstrates the power of anti-N IgG testing in seroepidemiologic studies to show real infection incidence rates in asymptomatic individuals, as discussed above.

In further analysis, anti-SARS-CoV-2 antibody persistence in different seropositive groups by the time since the last self-reported event was compared. The last self-reported event could not be the last as we found that around half (52.76 %) of the participants who did not self-report previous SARS-CoV-2 infection were anti-N IgG positive. In this study, anti-S IgG positivity remained high during 24 months since the last notified event in the hybrid and vaccination-induced immunity groups, whereas in the infection-induced immunity group, at some time points, anti-S positivity declined. It could be explained by the discussed tendency for short-lasting infection-induced immunity compared to vaccination-induced or hybrid immunity. The analysis of anti-N IgG persistence revealed interesting tendencies in the hybrid immunity group—a decline of anti-N IgG positivity within 9 months after the self-reported infection. The first decrease is noticed in month 6. A similar time and positivity-wise trend was reported previously [6]. However, the anti-N seropositivity shows no clear tendency at months 10–38 after the self-reported infection. The differences in anti-N seropositivity could be influenced by possible asymptomatic re-infections of SARS-CoV-2 during the Omicron period.

The advantage of our research is that this serosurvey of a population-based sample was performed in Spring 2023, the time the COVID-19 pandemic wanes. This study demonstrated different anti-SARS-CoV-2 IgG prevalence in infection, vaccination-induced, and hybrid immunity groups and revealed an unexpected age-related increase in anti-N IgG positivity in individuals with hybrid immunity. Furthermore, it was shown that anti-N IgG testing may be a valuable tool for the estimation of SARS-CoV-2 incidence rate in exposed yet asymptomatic individuals. Our study also confirmed previous observations of markedly declining anti-N positivity in 6–9 months post-infection. It should be mentioned that the results of our study had implications for planning vaccination strategies at a national level. Taking into account the high immunization rate of the general population, the policymakers focused the national vaccination campaign on risk groups including the elderly and individuals with chronic diseases [44].

Several limitations of our study design have to be named. Firstly, there was a relatively small sample size and the representativeness of the population. The minimum recommended sample size for the survey was 385. Our study sample size meets this requirement; however, the use of mixed methods for recruitment limits the representativeness and generalizability of the findings. The low participation rate and sampling challenges in COVID-19-related national-wide serosurveys are well known [22]. However, representativeness is not considered an essential factor in establishing the validity of the association between serological status and the incidence of infection [9]. Information about previous SARS-CoV-2 experience was taken from self-reported questionnaires. There is a risk of some incorrect information. However, self-reported vaccination information regarding dates of administration and vaccine details can be an effective surrogate when medical records are unavailable [45].

The study collected data in March–May 2023 at the end of Omicron variant predominance. Anti-N IgG positivity may have declined if infections occurred before Autumn 2022 as anti-N IgG as a marker of previous infection is valuable only for several months after the infection. This should be considered when estimating the prevalence of SARS-CoV-2 infections based on anti-N IgG positivity. Furthermore, we used semi-quantitative serological tests for the detection of anti-N and anti-S IgG, which classify the specimen as positive or negative according to specific cut-off. While similar tests are widely used in serosurveys, the cut-off values defining specimen positivity are different depending on the manufacturer. This should be taken into consideration when the results are compared between the serological studies. We categorized study participants into groups based on infection-induced, vaccination-induced, or hybrid immunity and assessed anti-SARS-CoV-2 seropositivity. However, this approach may not reflect the full complexity of immunity, as factors such as the timing of infections and vaccinations, and waning immunity are influencing antibody levels and are not explored here.

This study did not generate longitudinal antibody persistence data as the seropositivity duration estimation was based on data obtained in a single sampling. The duration of antibody persistence was estimated only by self-reported characteristics, including time after the antibody-inducing event. Although not providing longitudinal data, this study demonstrates a clear tendency for the establishment of humoral immunity in the analyzed population at the final phases of the COVID-19 pandemic.

## 5 Conclusions

Our study describes SARS-CoV-2 seroprevalence in a sample (N = 517) of a Lithuanian population-based study and supplements currently available scarce information on COVID-19 seroprevalence after the predominance of several SARS-CoV-2 Omicron subvariants. This study demonstrates high total seroprevalence in March–May 2023 in all age groups indicating a widely established antibody-mediated immunity against SARS-CoV-2 in Lithuania. Furthermore, the majority of study participants were shown to have hybrid immunity induced by vaccination and infection. This reflects high exposure to SARS-CoV-2 in Lithuania. Despite several limitations, the current seroprevalence study provides valuable information about the consequences of a prolonged COVID-19 pandemic period in Lithuania and suggests the formation of an established humoral immunity. The results presented here might be used for future decisions of public health policymakers.

## Ethics statement

The study complies with all regulations and written informed consent was obtained from the participants. The experiments were conducted according to established ethical guidelines. The study was approved by the Lithuanian Bioethics Committee on November 10, 2022 (approval No. L-22-09/1).

## Data availability statement

The authors confirm that the data supporting the findings of this study are available within the article and its supplementary materials.

## Funding

This study has received funding from the 10.13039/501100004504Research Council of Lithuania (LMTLT), agreement No. S-REP-22-18.

## CRediT authorship contribution statement

**Martynas Simanavičius:** Writing – review & editing, Writing – original draft, Visualization, Project administration, Methodology, Investigation, Formal analysis, Data curation, Conceptualization. **Indrė Kučinskaitė-Kodzė:** Writing – review & editing, Writing – original draft, Methodology, Investigation, Formal analysis, Conceptualization. **Snieguolė Kaselienė:** Writing – original draft, Methodology, Investigation, Formal analysis, Data curation. **Skirmantė Sauliūnė:** Methodology, Formal analysis, Data curation. **Dainius Gudas:** Investigation. **Ligita Jančorienė:** Resources, Project administration, Methodology. **Rūta Jasinskienė:** Software, Project administration. **Astra Vitkauskienė:** Resources, Project administration, Methodology. **Rasa Žūtautienė:** Software, Resources, Project administration. **Aurelija Žvirblienė:** Writing – review & editing, Supervision, Methodology, Data curation, Conceptualization. **Mindaugas Stankūnas:** Writing – review & editing, Writing – original draft, Supervision, Project administration, Methodology, Funding acquisition, Formal analysis.

## Declaration of competing interest

The authors declare the following financial interests/personal relationships which may be considered as potential competing interests:

Mindaugas Stankūnas reports financial support was provided by 10.13039/501100004504Research Council of Lithuania.
