# Supplementary material for: Prevalence of SARS-CoV-2-specific antibodies in a sample of the Lithuanian population-based study in Spring 2023
Source: Heliyon. 2024 Apr 12;10(8):e29343. doi: 10.1016/j.heliyon.2024.e29343 (PMC11053182; doi:10.1016/j.heliyon.2024.e29343)
Supplement: Multimedia component 1 [file mmc1.docx]

**THE QUESTIONNAIRE USED IN THE SURVEY**

"Prevalence of SARS-CoV-2-specific antibodies in a sample of the Lithuanian population-based study in Spring 2023"

1. Blood specimen ID code (with a letter, e.g. K-0001, V-0001)

2. The person arrived for the examination:

- having received an invitation by mail

- voluntarily (without a written personal invitation)

3. First name

4. Last name

5. Phone number (We will send the serological testing results to this number; if the phone number is not provided, the results will not be presented)

6. Gender

- Male

- Female

7. Date of birth

8. Place of residence

- City of Vilnius

- Vilnius district

- City of Kaunas

- Kaunas district

9. What is your highest level of education?

- Elementary (4 grades)

- Basic (10 grades)

- Secondary (12 grades)

- Specialized secondary (after completing a specialized secondary education program)

- Vocational (vocational school)

- Higher vocational (technical school, higher education institution)

- Higher non-university (college)

- Higher university (university, institute, academy)

- The person did not want to answer this question

10. What is your current occupation? (Multiple answer options can be selected)

- Employed

- Unemployed

- Student / Pupil

- On childcare leave

- Retired

- Disabled

- The person did not want to answer this question

11. What is your height?

Enter the number in centimeters, e.g. 168

12. What is your weight?

Enter the number in kilograms, e.g. 69

13. Have you been vaccinated against COVID-19 at least once?

- Yes

- No

- The person did not want to answer this question

14. How many times have you been vaccinated against COVID-19 in total?

- Once

- Twice

- Three times

- Four times

- I don't remember

- The person did not want to answer this question

15. Which COVID-19 vaccine(s) did you receive? (Multiple answer options can be selected)

- Comirnaty (Pfizer–BioNTech)

- Vaxzevria (AstraZeneca)

- Spikevax (Moderna)

- Janssen (Johnson & Johnson)

- I don't remember

- The person did not want to answer this question

16. When was the last time you were vaccinated with a COVID-19 vaccine? (If the person only remembers the year, enter the year in the "Other" field)

- 2021 Q1 (January, February, March)

- 2021 Q2 (April, May, June)

- 2021 Q3 (July, August, September)

- 2021 Q4 (October, November, December)

- 2022 Q1 (January, February, March)

- 2022 Q2 (April, May, June)

- 2022 Q3 (July, August, September)

- 2022 Q4 (October, November, December)

- 2023 Q1 (January, February, March)

- April 2023

- I don't remember

- The person did not want to answer this question

17. Have you had COVID-19?

- Yes, I had COVID-19 (confirmed or unconfirmed by a test)

- No, I didn't have it

- I don't know

- The person did not want to answer this question

18. How many times in total have you had COVID-19 confirmed by a PCR test?

- Never

- Once

- Twice

- Three times

- Four or more times

- I don't remember

- The person did not want to answer this question

19. How many times in total have you had COVID-19 confirmed by rapid antigen tests?

- Never

- Once

- Twice

- Three times

- Four or more times

- I don't remember

- The person did not want to answer this question

20. How many times in total have you had COVID-19 without confirmation by tests?

- Never

- Once

- Twice

- Three times

- Four or more times

- I don't remember

- The person did not want to answer this question

21. When was the last time you had COVID-19?

- 2020 Q1 (January, February, March)

- 2020 Q2 (April, May, June)

- 2020 Q3 (July, August, September)

- 2020 Q4 (October, November, December)

- 2021 Q1 (January, February, March)

- 2021 Q2 (April, May, June)

- 2021 Q3 (July, August, September)

- 2021 Q4 (October, November, December)

- 2022 Q1 (January, February, March)

- 2022 Q2 (April, May, June)

- 2022 Q3 (July, August, September)

- 2022 Q4 (October, November, December)

- 2023 Q1 (January, February, March)

- April 2023

- I don't remember

- The person did not want to answer this question

22. When you last had COVID-19, did you experience any symptoms typical of the disease?

- Yes, I felt symptoms and was hospitalized because of them

- Yes, I felt symptoms, but I treated myself at home

- No, I did not experience any symptoms

- I don't remember

- The person did not want to answer this question

23. When you last had COVID-19, which of the listed symptoms did you experience?

| Symptoms | Yes | No | I don’t remember |
| --- | --- | --- | --- |
| Fever |  |  |  |
| Sore throat, hoarseness |  |  |  |
| Muscle pain |  |  |  |
| Stuffy nose |  |  |  |
| Cough |  |  |  |
| Loss of smell or taste |  |  |  |
| Severe fatigue |  |  |  |
| Diarrhea |  |  |  |
| Nausea or vomiting |  |  |  |
| Increased breathing rate |  |  |  |
| Other symptoms |  |  |  |
| Shortness of breath |  |  |  |

24. If you indicated in the previous question that you experienced other symptoms, please specify which ones.

25. Have your close relatives living with you had a confirmed COVID-19 infection (confirmed by a PCR or rapid antigen test)?

- Yes

- No

- I don't know

- The person did not want to answer this question

26. Do you have any chronic diseases?

- Yes

- No

- The person did not want to answer this question

27. Which chronic diseases do you have? (Select all applicable answer options)

- Cardiovascular diseases

- Diabetes

- Oncological diseases

- Respiratory system diseases

- Kidney diseases

- Autoimmune diseases (e.g., rheumatic diseases, autoimmune thyroiditis, multiple sclerosis, psoriasis, etc.)

- Allergic diseases

- The person did not want to answer this question

28. Are you currently taking immunosuppressive drugs (e.g., chemotherapy, biological therapy, immunotherapy, etc.)?

- Yes

- No

- I don't know

- The person did not want to answer this question
