# Supplementary material for: Prevalence of SARS-CoV-2-specific antibodies in a sample of the Lithuanian population-based study in Spring 2023
Source: Heliyon. 2024 Apr 12;10(8):e29343. doi: 10.1016/j.heliyon.2024.e29343 (PMC11053182; doi:10.1016/j.heliyon.2024.e29343)
Supplement: Multimedia component 2 [file mmc2.docx]

SUPPLEMENTARY:

**Table S1. Description of study sample groups by COVID-19 vaccination status.**

| **Characteristics** | **Total**  **(%/n)**  **(N=517)** | **Self-reported SARS-CoV-2 infection**  **(%/n)**  **(N=318)** | **COVID-19 vaccination (%/n)** | | **P-value** |
| --- | --- | --- | --- | --- | --- |
|  |  |  | ***Vaccinated***  ***(N=457)*** | ***Not vaccinated***  ***(N=60)*** |  |
| **Sex** | | | | | |
| Males | 32.5 / 168 | 29.6 / 94 | 32.6 / 149 | 31.7 / 19 | 1.000^a^ |
| Females | 67.5 / 349 | 70.4 / 224 | 67.4 / 308 | 68.3 / 41 |  |
| **Age** | | | | | |
| Mean ± SD | 51.75 ± 15.80 | 49.40 ± 15.72 | 51.65 ± 16.06 | 52.51 ± 13.74 | 0.826^b^ |
| 18 – 39 yr. | 25.0 / 129 | 29.9 / 95 | 25.2 / 115 | 23.3 / 14 | 0.869 |
| 40 - 64 yr. | 52.6 / 272 | 52.2 / 166 | 52.7 / 241 | 51.7 / 31 |  |
| 65+ yr. | 22.4 / 116 | 17.9 / 57 | 22.1 / 101 | 25.0 / 15 |  |
| **Municipality** | | | | | |
| Vilnius city | 48.7 / 252 | 49.4 / 157 | 48.6 / 222 | 50.0 / 30 | 0.489 |
| Vilnius district | 10.1 / 52 | 29.9 / 95 | 9.4 / 43 | 15.0 / 9 |  |
| Kaunas city | 28.6 / 148 | 10.1 / 32 | 29.3 / 134 | 23.3 / 14 |  |
| Kaunas district | 12.6 / 65 | 10.7 / 34 | 12.7 / 58 | 11.7 / 7 |  |
| **Education** | | | | | |
| Secondary or lower | 24.6 / 127 | 21.7 / 69 | **22.8 / 104*** | **38.3 / 23** | **0.013** |
| Higher | 26.7 / 138 | 26.4 / 84 | 26.5 / 121 | 28.3 / 17 |  |
| University | 48.7 / 252 | 51.9 / 165 | **50.8 / 232*** | **33.3 / 20** |  |
| **Employment** | | | | | |
| Employed | 69.6 / 360 | 74.2 / 236 | **71.3 / 326** | **56.7 / 34** | **0.025** ^a^ |
| Unemployed | 30.4 / 157 | 25.8 / 82 | **28.7 / 131** | **43.3 / 26** |  |
| **BMI** | | | | | |
| Mean ± SD | 26.29 ± 4.71 | 25.89 ± 4.56 | 26.31 ± 4.69 | 26.15 ± 4.93 | 0.764^b^ |
| <25 | 44.6 / 230 | 47.8 / 152 | 44.4 / 203 | 45.8 / 27 | 0.499 |
| 25-29.99 | 34.7 / 179 | 34.0 / 108 | 35.4 / 162 | 28.8 / 17 |  |
| 30> | 20.7 / 107 | 18.2 / 58 | 20.1 / 92 | 25.4 / 15 |  |
| **Chronic diseases** | | | | | |
| No | 53.1 / 274 | 53.5 / 170 | 54.2 / 247 | 45.0 / 27 | 0.181 ^a^ |
| Yes: | 46.9 / 242 | 46.5 / 148 | 45.8 / 209 | 55.0 / 33 |  |
| Cardiovascular | 59.1 / 143 | 57.4 / 85 | 59.8 / 125 | 54.5 / 18 | 0.573 ^a^ |
| Diabetes | 8.7 / 21 | 6.8 / 10 | 8.1 / 17 | 12.1 / 4 | 0.502 ^a^ |
| Autoimmune | 8.7 / 21 | 9.5 / 14 | 7.7 / 16 | 15.2 / 5 | 0.179 ^a^ |
| Respiratory system | 7.9 / 19 | 7.4 / 11 | **6.2 / 13** | **18.2 / 6** | **0.030** ^a^ |
| Oncological | 6.2 / 15 | 4.7 / 7 | 5.7 / 12 | 9.1 / 3 | 0.438 ^a^ |
| Kidney | 2.9 / 7 | 4.1 / 6 | 2.4 / 5 | 6.1 / 2 | 0.244 ^a^ |
| **Use of immunosuppressive drugs** | | | | | |
| Yes | 3.1 / 16 | 3.8 / 12 | 2.8 / 13 | 5.0 / 3 | 0.415 ^a^ |
| No | 86.9 / 501 | 96.2 / 306 | 97.2 / 444 | 95.0 / 57 |  |

* P < 0.05 compared to not vaccinated (z-test); ^a^ - Fisher's Exact Test; ^b^ - Mann-Whitney U Test; BMI – body mass index; P value < 0.05 is shown in bold.

**Table S2. Comparison of self-reported cases of SARS-CoV-2 infection and the vaccination status.**

| **Characteristics** | **COVID-19 vaccination status (%/n)** | | **Total**  **(%/n)**  **(N=517)** | **P-value^k^** |
| --- | --- | --- | --- | --- |
|  | ***Vaccinated***  ***(N=457)*** | ***Not vaccinated***  ***(N=60)*** |  |  |
| **Previous SARS-CoV-2 infection** | | | | |
| Yes | 63.9 / 276 | 72.4 / 42 | 64.9 / 318 | 0.242 |
| No | 36.1 / 156 | 27.6 / 16 | 35.1 / 172 |  |
| **Previous SARS-CoV-2 infection confirmed by PCR test** | | | | |
| None | 57.8 / 264* | 40.0 / 24 | 55.7 / 288 | **<0.001** |
| 1 time | 37.0 / 169 | 43.3 / 26 | 37.7 / 195 |  |
| 2 and more times | 5.3 / 24* | 16.7 / 10 | 6.6 / 34 |  |
| **Previous SARS-CoV-2 infection confirmed by a rapid antigen test** | | | | |
| None | 68.5 / 313 | 76.7 / 46 | 69.4 / 359 | 0.133 |
| 1 time | 29.1 / 133 | 18.3 / 11 | 27.9 / 144 |  |
| 2 and more times | 2.4 / 11 | 5.0 / 3 | 2.7 / 14 |  |
| **Previous SARS-CoV-2 infection not confirmed by any test.** | | | | |
| None | 86.4 / 395* | 71.7 / 43 | 84.7 / 438 | **0.006** |
| 1 time | 10.3 / 47 | 18.3 / 11 | 11.2 / 58 |  |
| 2 and more times | 3.3 / 15* | 10.0 / 6 | 4.1 / 21 |  |
| **Any symptoms during the last SARS-CoV-2 infection** | | | | |
| No | 4.3 / 12 | 4.8 / 2 | 4.4 / 14 | 1.000 |
| Yes: | 95.7 / 264 | 95.2 / 40 | 95.6 / 304 |  |
| Fever | 74.6 / 194 | 77.5 / 31 | 75.0 / 225 | 0.845 |
| Fatigue | 68.7 / 180 | 77.5 / 31 | 69.9 / 211 | 0.355 |
| Runny nose | 60.5 / 156 | 62.5 / 25 | 60.7 / 181 | 0.863 |
| Sore throat | 61.1 / 157 | 50.0 / 20 | 59.6 / 177 | 0.225 |
| Cough | 55.4 / 143 | 67.5 / 27 | 57.0 / 170 | 0.172 |
| Muscle pain | 50.4 / 130 | 62.5 / 25 | 52.0 / 155 | 0.176 |
| Loss of taste or smell | 35.1 / 92 | 35.9 / 14 | 35.2 / 106 | 1.000 |
| Frequent breathing | 22.2 / 57 | 39.5 / 15 | 24.4 / 72 | **0.026** |
| Shortness of breath | 17.6 / 46 | 37.5 / 15 | 20.2 / 61 | **0.006** |
| Nausea or vomiting | 11.1 / 29 | 15.4 / 6 | 11.6 / 35 | 0.425 |
| Diarrhea | 7.3 / 19 | 29.7 / 11 | 10.1 / 30 | **<0.001** |
| Other | 10.9 / 28 | 7.5 / 3 | 10.4 / 31 | 0.780 |
| **Previous SARS-CoV-2 infection confirmed to other household members.** | | | | |
| Yes | 51.7 / 234 | 51.7 / 31 | 51.7 / 265 | 1.000 |
| No | 48.3 / 219 | 48.3 / 29 | 48.3 / 248 |  |

* P < 0.05 compared to not vaccinated (z-test); k - chi-squared test (three or more groups) or Fisher exact test (two groups); P < 0.05 is shown in bold.

**Table S3. Comparison of SARS-CoV-2 vaccination and infection status with the determined anti-SARS-CoV-2 IgG positivity.**

| **Characteristics** | **Anti-N IgG**  **(%/n)** | | | **Anti-S IgG**  **(%/n)** | | |  |
| --- | --- | --- | --- | --- | --- | --- | --- |
|  | ***Positive***  ***(N=300)*** | ***Negative***  ***(N=217)*** | ***P-value^k^*** | ***Positive***  ***(N=501)*** | ***Negative***  ***(N=16)*** | ***P-value^k^*** |  |
| **Sex** | | | | | | |  |
| Males | 54.2 / 91 | 45.8 / 77 | 0.254 | 95.2 / 160 | 4.8 / 8 | 0.173 |  |
| Females | 59.9 / 209 | 40.1 / 140 |  | 97.7 / 341 | 2.3 / 8 |  |  |
| **Age** | | | | | | |  |
| 18 – 39 yr. | 52.7 / 68 | 47.3 / 61 | 0.352 | 96.1 / 124 | 3.9 / 5 | 0.757 |  |
| 40 - 64 yr. | 60.3 / 164 | 39.7 / 108 |  | 97.4 / 265 | 2.6 / 7 |  |  |
| 65+ yr. | 58.6 / 68 | 41.4 / 48 |  | 96.6 / 112 | 3.4 / 4 |  |  |
| **Vaccination status** | | | | | | | |
| Yes | 56.7/ 259 | 43.3 / 198 | 0.096 | **99.8 / 456** | **0.2 / 1** | **<0.001** |  |
| No | 68.3 / 41 | 31.7 / 19 |  | **75.0 / 45** | **25.0 / 15** |  |  |
| **Number of received COVID-19 vaccine doses** | | | | | | | |
| 1 | **83.3 / 10** | **16.7 / 2^a^** | **0.036** | 100.0 / 12 | 0.0 / 0 | 0.492 |  |
| 2 | **61.9 / 83** | **38.1 / 51^ab^** |  | 99.3 / 133 | 0.7 / 1 |  |  |
| 3 | **55.1 / 151** | **44.9 / 123^bc^** |  | 100.0 / 274 | 0.0 / 0 |  |  |
| 4 | **41.7 / 15** | **58.3 / 21^c^** |  | 100.0 / 36 | 0.0 / 0 |  |  |
| **COVID-19 vaccine** | | | | | | | |
| Comirnaty | 55.8 / 159 | 44.2 / 126 | 0.449 | **100.0 / 285^a^** | **0.0 / 0^a^** | **0.002** |  |
| Vaxzevria | 69.6 / 16 | 30.4 / 7 |  | **95.7 / 22^b^** | **4.3 / 1^b^** |  |  |
| Spikevax | 73.3 / 11 | 26.7 / 4 |  | 100.0 / 15 | 0.0 / 0 |  |  |
| Janssen | 50.0 / 6 | 50.0 / 6 |  | 100.0 / 12 | 0.0 / 0 |  |  |
| Vaxzevria + Comirnaty | 51.9 / 41 | 48.1 / 38 |  | 100.0 / 79 | 0.0 / 0 |  |  |
| Other | 62.5 / 20 | 37.5 / 12 |  | 100.0 / 32 | 0.0 / 0 |  |  |
| **Previous SARS-CoV-2 infection** | | | | | | | |
| Yes | **61.3 / 195** | **38.7 / 123** | **0.035** | 97.5 / 310 | 2.5 / 8 | 0.286 |  |
| No | **51.2 / 88** | **48.8 / 84** |  | 95.3 / 164 | 4.7 / 8 |  |  |
| **Previous SARS-CoV-2 infection confirmed by PCR test** | | | | | | | |
| None | 55.6 / 160 | 44.4 / 128 | 0.126 | 96.5 / 278 | 3.5 / 10 | 0.851 |  |
| 1 time | 59.0 / 115 | 41.0 / 80 |  | 97.4 / 190 | 2.6 / 5 |  |  |
| 2 and more times | 73.5 / 25 | 26.5 / 9 |  | 97.1 / 33 | 2.9 / 1 |  |  |
| **Previous SARS-CoV-2 infection confirmed by a rapid antigen test** | | | | | | | |
| None | **55.2 / 198^a^** | **44.8 / 161^a^** | **0.033** | 95.8 / 344 | 4.2 / 15 | 0.099 |  |
| 1 time | 62.5 / 90 | 37.5 / 54 |  | 99.3 / 143 | 0.7 / 1 |  |  |
| 2 and more times | **85.7 / 12^b^** | **14.3 / 2^b^** |  | 100.0 / 14 | 0.0 / 0 |  |  |
| **Previous SARS-CoV-2 infection not confirmed by any test.** | | | | | | | |
| None | 56.8 / 249 | 43.2 / 189 | 0.320 | 96.8 / 424 | 3.2 / 14 | 0.751 |  |
| 1 time | 67.2 / 39 | 32.8 / 19 |  | 98.3 / 57 | 1.7 / 1 |  |  |
| 2 and more times | 57.1 / 12 | 42.9 / 9 |  | 95.2 / 20 | 4.8 / 1 |  |  |
| **Any symptoms during the last SARS-CoV-2 infection** | | | | | | | |
| Yes | 62.2 / 189 | 37.8 / 115 | 0.167 | 97.4 / 296 | 2.6 / 8 | 1.000 |  |
| No | 42.9 / 6 | 57.1 / 8 |  | 100.0 / 14 | 0.0 / 0 |  |  |
| **Previous SARS-CoV-2 infection confirmed in other household members** | | | | | | | |
| Yes | **62.6 / 166** | **37.4 / 99** | **0.032** | 98.1 / 260 | 1.9 / 5 | 0.128 |  |
| No | **53.2 / 132** | **46.8 / 116** |  | 95.6 / 237 | 4.4 / 11 |  |  |

abc - different letters show a statistically significant difference (P < 0.05); k - chi-squared test (three or more groups) or Fisher exact test (two groups); P value < 0.05 is shown in bold.

**Fig. S1. The step-by-step procedures of the study.**


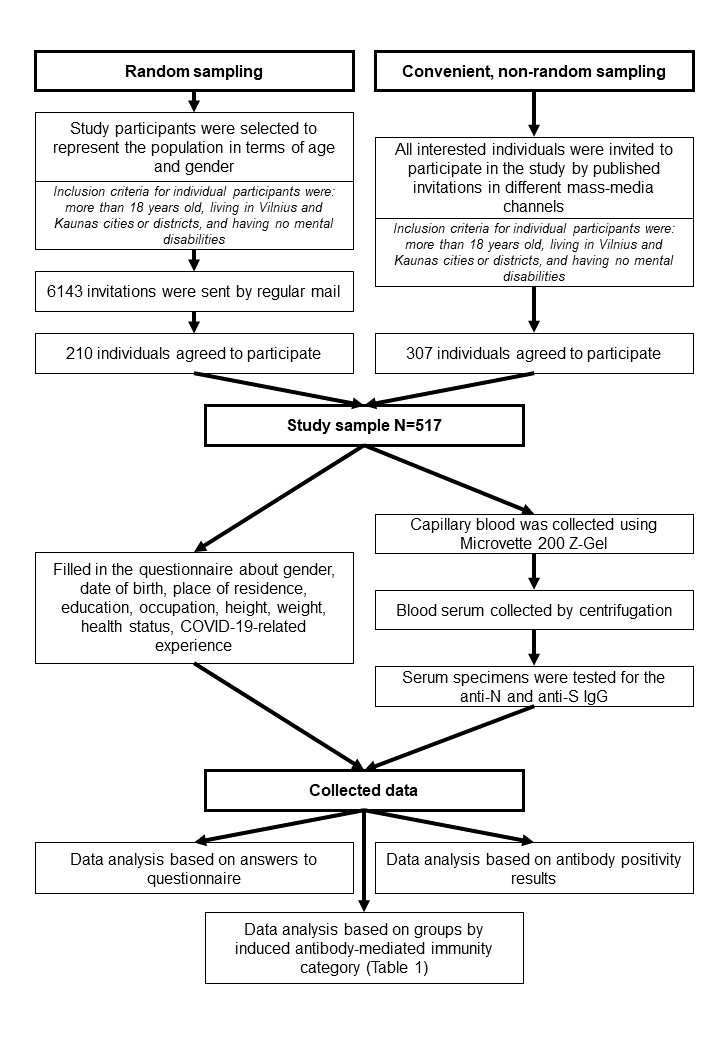


**
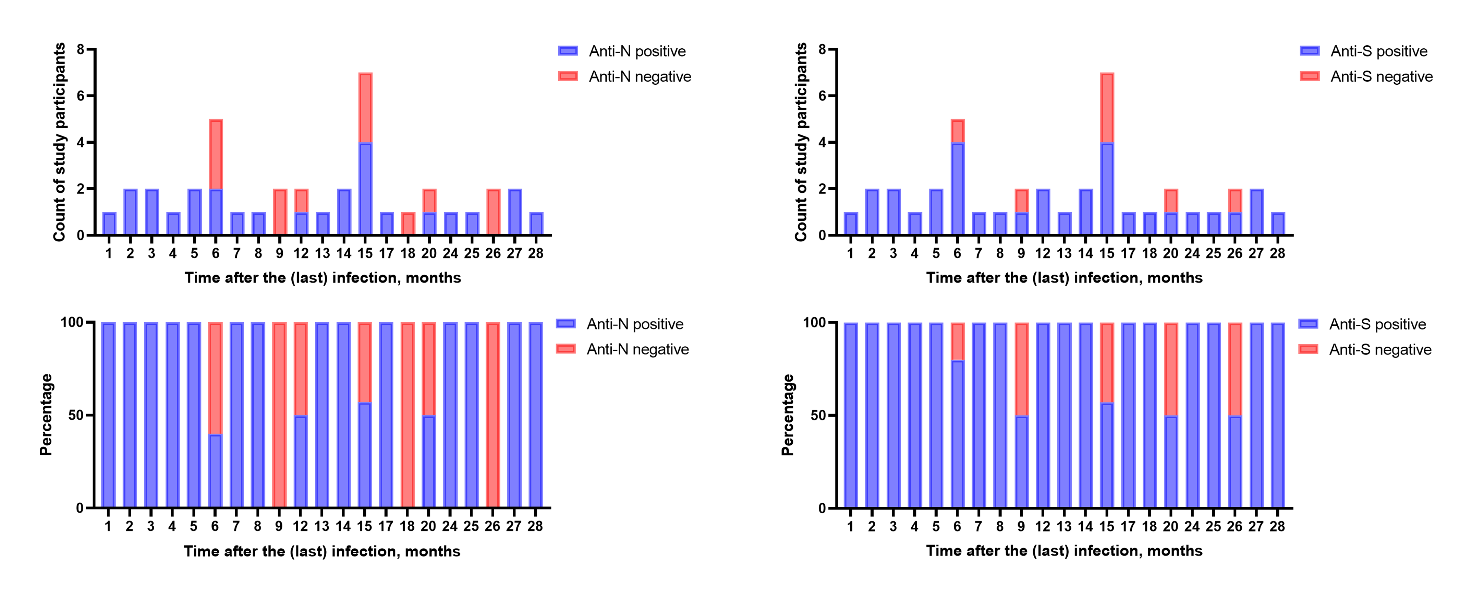
**

**Fig.S2. Anti-SARS-CoV-2 antibody persistence in participants with only infection-induced humoral immunity.**

**
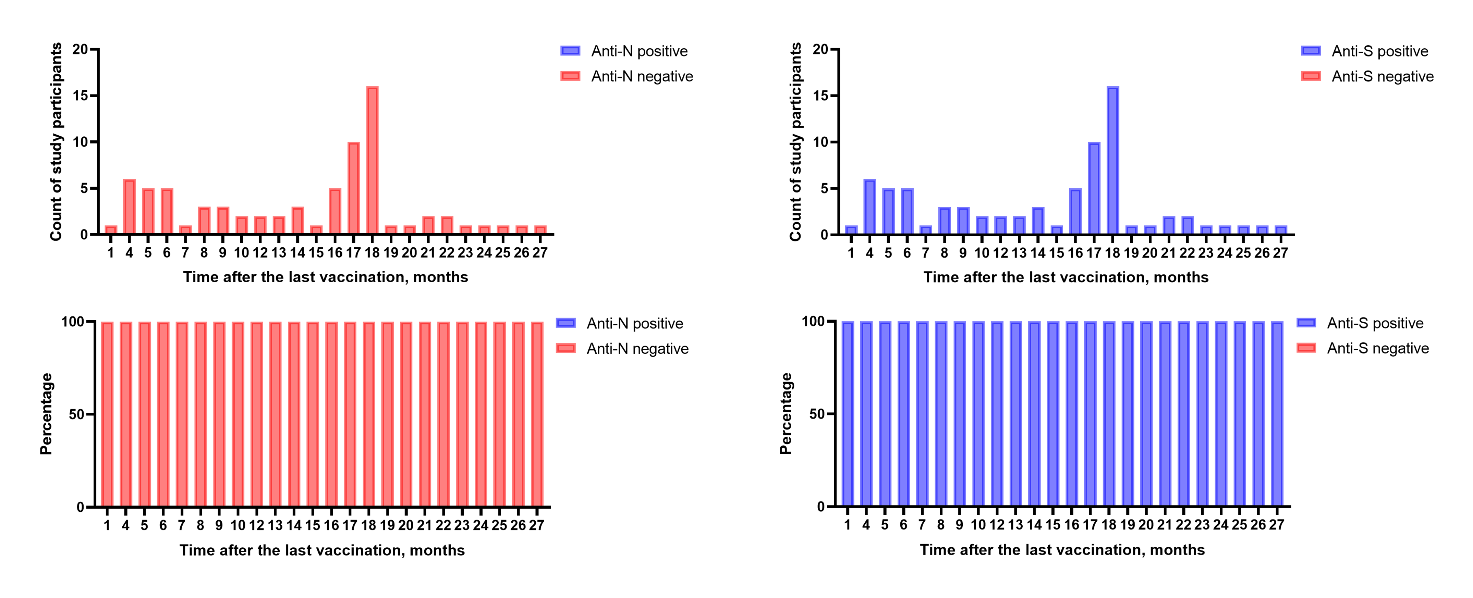
**

**Fig.S3. Anti-SARS-CoV-2 antibody persistence in participants with only vaccination-induced humoral immunity.**

**
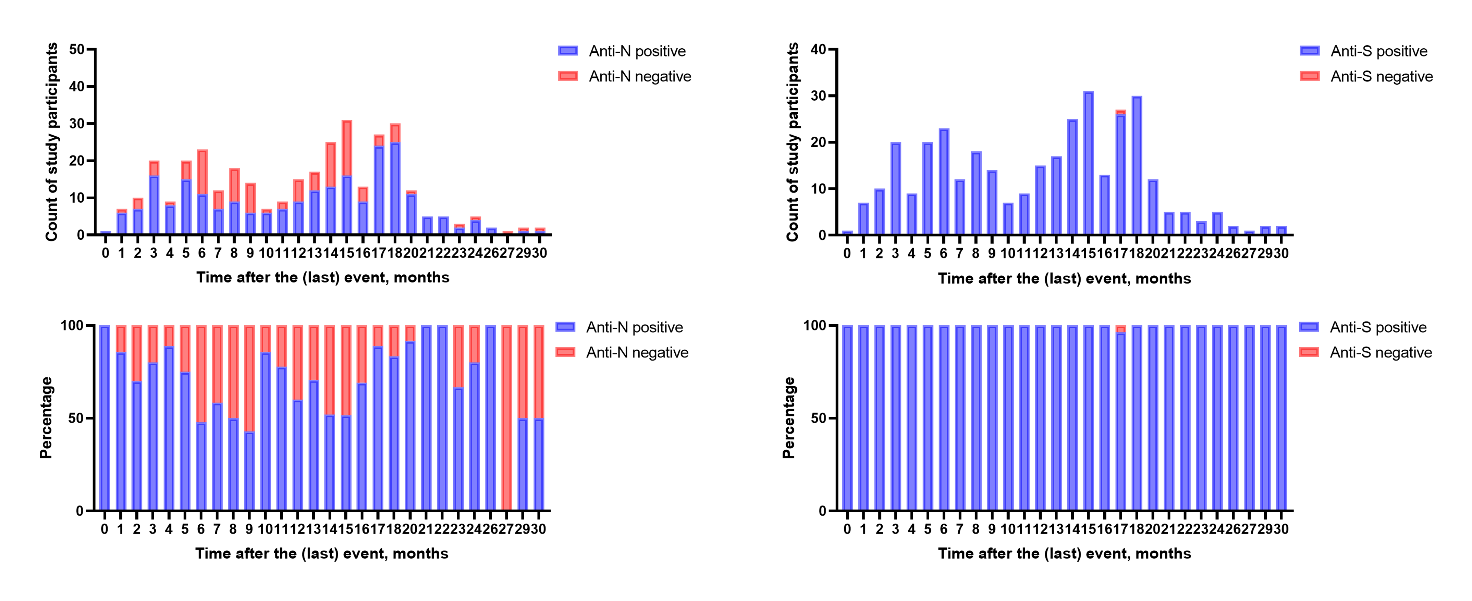
**

**Fig.S4. Anti-SARS-CoV-2 antibody persistence in participants with hybrid humoral immunity. No time points are excluded compared to Figure 2.**
